# Inaugural Address

**Published:** 1894-01

**Authors:** L. D. Shepard

**Affiliations:** President of the World's Columbian Congress.


THE
AMERICAN JOURNAL
—OF—
DENTAL SCIENCE.

VOL. XXVII. BALTIMORE, JANUARY, 1894. NO. 9.

ARTICLE I.

### INAUGURAL ADDRESS.

BY L. D. SHEPARD, A. M., D. D. S., D. M. D.
President of the World's Columbian Congress.

It is becoming that the gathering of the nations in this wonderfully energetic metropolis, in this memorable year for America, should be taken advantage of for the holding of an Exposition of Dentistry, of all countries, in all branches, scientific and practical, as it exists to day.

The origin of the Congress dates from action taken three years ago by the two governing dental societies of the country—action which anticipated, I believe, the organization of the World's Congress Auxiliary with its high aims and broad plans. We were happy to ally ourselves with this Auxiliary; and so participate in the successful administration of its high educational result. While our autonomy has been preserved, we are proud to be one of the many Congresses held under its auspices for the good of society.

The two societies jointly created a trust, by the appointment of a General Executive Committee, and agreed

"that any action that this committee may take shall be final and binding."

The only instruction to the committee in what might be called its charter was "to take such action as in its judgment it may deem best for creating an organization for the purpose of holding a dental meeting in Chicago in 1893, which the reputable dentists throughout the world shall be invited to attend."

It was understood that this organization should be *sui generis*, complete in itself without continued corporate existence. The committee accepted the trust as a great and responsible one, and has worked assiduously to justify the confidence reposed in it. It has ever endeavored to do the best thing and to be just to all.

The committee understood that the primal and fundamental object was to hold a dental meeting so broad and catholic in spirit and policy that it should be essentially inclusive of everything good and exclusive of none, to the end that all creeds may be heard and the truth through a multitude of counsel be evolved.

In accordance with this conception the invitation to membership was so broad as to include every dentist in the world who was considered respectable and honorable. The only conditions were that he be a legal practitioner in his country and carry on his practice in accordance with recognized ethical principles. The dentist of affluence whose clientele is most aristocratic has no more right here than the humblest of those who honor our vocation by good work in the remotest country village.

Perhaps of no other profession of equal altitude and amplitude can it be said, as of dentisrty, that its evolution is embraced within the span of one human life.

There are men living to-day, still active and officers of this Congress, who were in practice in the day of small things of our profession. They are with us, witnesses of the marvelous march of progress; veteran survivors of the noble army of investigators, inventors, authors, teachers.

and honorable workers, who, in various spheres, with diversified talents, with patient toil, with fidelity to present duties, and with hope and faith were achieving the victory which we celebrate to-day.

Other professions and other branches of the great healing art have made great strides in the same space of time, but of no other vocation or profession devoted to human happiness and amelioration, except, perhaps, photography and applied electricity, can it be said with equal truth, that for system and well rounded development such a contrast can be shown between the humble acquirements and achievements of the youth of the men referred to and the ripened conditions existing to-day. Medicine, unlike mathematics, is not an exact science, but of all branches of surgery there is probably none in which the realization of the ideal is so nearly secured as in dentistry.

All vocations are entitled to honor where the work is honest and the ends sought for are commendable, but especial honor has ever been rendered to those vocations which deal with human ailments and sufferings, whether mental or physical, and to such vocations from antiquity the differential honor of the name profession, formerly more narrowly used than at present, has been given. The relative rank among professions is not unimportant, but can we not claim with proper modesty and truthfulness that for the prevention and amelioration of human suffering, and for the peace, health, and prolongation of human existence, dentistry is to-day without a peer among them all?

It has been alloted to others to present to you the latest phases of every department of professional study, scientific, practical, and humanitarian, and to demonstrate the knowledge and skill in theory and practice. The ablest men in dentistry in the world will bring the products of their brains and hands before you. The week will be full to overflowing with interesting and valuable papers and discussions, with brilliant lantern illustrations of the patient and self-sacrificing investigations of men truly scientific who have delved into the mysteries of cell-life, and will bring before our eyes the great unseen, wherein lies the foundation of all etiology and scientific knowledge and progress. Others with skillful hands working in harmony with active brains, will demonstrate the manifold practical applications of theory and experience for our better equipment for the daily duties of the office.

In presenting to you this inaugural address, my duty, it seems to me, should be, not to anticipate or encroach upon the work of these others, but to attempt to give a brief outline of the evolution of the profession, with the salient points which mark its progress.

That dentistry to some extent is an ancient art cannot be questioned, but so meager are the references to it, in what remains that is authentic, that we may dismiss it from consideration at this time with the remark that nothing of value has come down to us from antiquity. The same is true of all the past up to the century preceding this. In fact, even as an art we may consider dentistry as modern, while as a science it is altogether modern.

Time will not permit me to review at length what is known of its condition in the last century, nor shall I refer but briefly to the conditions in the early part of the present century. The very valuable report which will be made by the Committee on History with so many co-laborers will, I have no doubt, give us a very full history, for the first time, of early dentistry in this country. This Congress would be well worth its cost if no other result should come of it but this.

There are no sharp lines of demarcation in evolutionary processes, and in their review we find the changes to be so gradual and long-continued that it is difficult to fix upon a time which is so distinctly marked as to be called a natal day. We have been accustomed to date the birth of a man from his advent upon the visible stage, and yet that day witnesses but a change of environment, and, as a large proportion of humanity believe, the birth of the soul dating months before. So dentistry had its embryo stage; its inception is shadowed by the mists of antiquity. Through ages it slowly gained, and we cannot describe its progress. During the last century and in the early part of this century there were signs of life and movement, the quickening had taken place, the world was expectant, and the joyful consummation, by the birth of a new profession, freighted with beneficence to suffering humanity, occurred in 1839, in the city of Baltimore, by the organization of the first dental college in the world. As a scientific profession that is its natal day. There had been life before, as in the case of the man, but the environment had so changed that now there was a new air to breathe, new sources of nutrition, the barrier of the previous restricted environment was removed, and there was free chance for growth to the stature of the full typal ideal.

Let us not forget to hold in the highest honor the devoted men who assisted at this birth. They are not the fathers of scientific dentistry—the primal causes date farther back—but their care, their oversight and efficient ministrations, in guiding and assisting in the culmination of evolutionary changes at its critical period, were most creditable. Like the Great Physician, they thought less of self than of humanity. They brought into the world the good "Evangel" of dentistry, for previous to that time knowledge and skill were guarded and sold as private property, while that day heralds the advent of "good tidings" to every man in search of knowledge for the good of men. The dental aspirant before that time found every avenue to knowledge carefully defended. Knowledge could only be obtained in the private office of a dentist, and the ambitious student was obliged to buy it frequently at fabulous prices prohibitive to the majority. The dentist who had obtained reputation thus received considerable revenue from such seekers—and in time exacted a promise of the student to likewise guard the information imparted.

The Baltimore College of Dental Surgery was the first Thesaurus of dentistry. Here were first deposited the stores of knowledge which were before in individual keeping. Here, too, also for the first time in dentistry, for a stated moderate price, the student could draw from this treasure-house all the accumulated knowledge it possessed.

Let us bear in mind that the prominence given to the establishment of this first professional school, as marking a natal period, is not primarily because it was the first professional school; that is of course noteworthy, so is the establishment of a new manufacturing business in a community, for it may be followed by other enterprises, and so the place become prosperous—but the point should be emphasized, and it is the greatest point, that here was the beginning of a change of *spirit* as well as of method. Before this it was a trade, ever mindful of self, accumulative, afraid of competition, exclusive, faithful to the immediate patient and anxious to do him good, but regardless of the rest of the world, dominated by selfish interests, carefully hoarding knowledge, with no broad professional spirit—no brotherhood of feeling.

It is hard to realize at this day when there is such freedom—such fraternal feeling, and actual competition to impart knowledge—that such a contrast in professional spirit could have existed six decades ago. It *is* a fact, however, and there are members of this congress who commenced their study of dentistry in the old way. In my opinion this change of spirit is at the foundation of the new era, and all progress since then may be said to rest upon it.

With similar prophetic vision and patriotic motive in this same epochal year, the great handmaid and co-laboror of the college was established by the publication of the first dental periodical in the world, the AMERICAN JOURNAL OF DENTAL SCIENCE. The same spirit—the same ambition for the profession and the same regard for humanity—actuated the generous and enthusiastic founders of journalism as of the college.

As human nature is constituted, the college and the magazine are not enough to redeem and enlighten. A few are reached by these agencies, but to stir the great mass, such is the inertia of ignorance and routine, the magnetism of personal intercourse is needed. That the succeeding year, 1840, should witness the supplying of this requisite, is another proof of the vivifying forces operating at this period.

The same few pioneers of progress who started the college and magazines in 1839, in association with others of like spirit and motive, on August 18th, 1840, met in New York and organized the American Society of Dental Surgeons.

No authentic records of any previous dental society exist. So this society may be respected as the prototype of the multitude of societies which since then can justly claim so great a share in the growth in which we rejoice to-day.

In the organization of this society, the resolution offered by Prof. Chapin A. Harris, for the appointment of a committee to draft a Constitution and By-Laws, commenced with these words, which have proved prophetic :

"*Resolved*, That it is the opinion of this convention that the science of dental surgery should be advanced and the interests of all well-informed practitioners and the community at large be promoted by the formation of a National Society of Dentists."

And in the constitution which was adopted, the first article struck the key-note for all time in these words: "The objects of this Society are to promote union and harmony among all respectable and well-informed Dental Surgeons; to advance the science by free communication and interchange of sentiments, either written or verbal, between members of the Society, both in this and other countries; in fine, to give character and respectability to the profession," etc.

We thus see, within a few months, the erection of the great tripod upon which all professional advancement must rest—the college, the journal, and the association.

The first aimed to thoroughly prepare the novitate for the highest usefulness, while the broad field of the others was the great mass of practitioners of varied proficiency.

Dr. C. W. Ballard, of New York, in an essay describing the profession at this period, says it might be divided into three classes:

The first class, embracing about one-half of all in practice, "consisted of those whose ignorance was their only excuse for the injuries they inflicted upon their patients, and ultimately their profession, as also of those who, having purchased or traded for a secret or two, depended upon bold-faced and unblushing impudence for their success. * * * Such men could only stand in their own estimation by dragging the profession down to their own level. * * * Dentists, to this day, suffer to some extent from the odium brought upon their calling by the acts of these men. Dentists of this class know little, and cared less, about the duties devolving upon them, and yet they were always ready to receive pupils and instruct them in the secrets and mysteries of dental science, provided they were well paid for it. The fees exacted in these cases varied from five dollars to one thousand. * * * The length of time occupied by these initiatory proceedings depended very much upon the ability of the student and the ignorance of the teacher. It was generally conceded by these dentists that the shorter the time wasted in this manner the better for all parties."

The second class, embracing about three-eights of the whole number, "may be considered as consisting of those dentists who, having obtained as great a knowledge of the principles and practice of dental surgery as their time, means, or opportunities would allow, came at once to the conclusion that so long as they did the best they knew how for their patients, and comported themselves in other respects as became good citizens, they had done their whole duty. * * * With these may be included those who commenced with little or no education, and were compelled, in order to compete with those around them, to add, by every meaning in their power, to the knowledge and experience that their practice was daily giving them. Many of these men eventually became, to a certain extent, good practitioners, but of the best of them it would be difficult to say whether the good or the evil which they had done in their day preponderated. Dentists of the second class were much better acquainted with their professional duties than those first described, and very many of them excelled in that branch of the practice known as mechanical dentistry; and, in justice to them, it must be borne in mind that, at the time of which we are writing, mechanical dentistry was considered, by a majority of the profession, to be by far the most important part of dental practice."

Those of the third class, about one-eighth of all, a good proportion of whom were medically educated, "had, as a result of their course of practice and deportment generally, acquired far more reputation and influence. * * * These few men seemed, from the outset, to have been impressed with the belief that the resources of the science were by no means developed—that dental surgery held a position far beneath that to which it was entitled * * * and that, as all these evils could and should be remedied, it was their duty to devote a portion of their time and energies to the work."

It will be found that this latter class, numerically so small, was the leaven which was to redeem the whole mass. The same law held here, as in material things, that the quality of the leaven was more important than the quantity.

In the year 1841 was enacted the first State law in regard to dentistry. It is probably also the first law in any country. I have not included legislation as among the important and fundamental causes of dental progress, for the reason that the Alabama law stood alone among the States in this country for over twenty-five years, the next law to be passed being that of New York in 1868. The English law was enacted in 1878, and in other countries about that date or later. While an indirect and inevitable result of dental laws has been to help the colleges by making an education a pre-requisite to legal admission to practice, and hence to elevate the standards of the profession, there could be no justification for such prohibitive or restrictive enactments except as a safeguard to public health under the general police powers which in modern times have become so broadly applied and universally supported by the judiciary.

The struggle to secure these laws has been long and hard. Many efforts have proved repeatedly unsuccessful, but opposition has been overcome until at the present date nearly every State and country has such laws, identical in object and essential features, and varying only in what might be called minor details. There are few now who doubt their justice and usefulness.

In securing their enactment the profession has generally taken a leading part, and it can be truthfully claimed that the motive has not been selfish but philanthropic.

The next event in dental history was so brilliant as to be worthy of being called the most notable and beneficent discovery of the century or of all centuries—anæsthesia. What discovery or invention is compared to this, by which "the knife of the surgeon is steeped in the waters of forgetfulness and the deepest furrow in the knotted brow of agony is forever smoothed away," to quote the poetic words of the venerable, but still youthful author of the term anesthesia, Oliver Wendell Holmes? While there has been an ether controversy, there cannot be an anesthesia controversy. The ether controversy was waged with great earnestness and bitterness, but with the lapse of time and the removal of those directly interested, the credit is now generally given to the late Dr. Morton, a dentist of

Boston. He it was who took his life in his hands, and, with sublimate courage or audacity, put in jeopardy human life to solve the problem of anesthesia with ether. He traveled in darkness an unknown road; he succeeded, and demonstrated to a skeptical world anesthesia by etherization. The Massachusetts General Hospital justly and elegantly expressed the sentiments of mankind in its inscription upon the present given him in the words: "He has become poor in a cause which has made the world his debtor."

Without detracting from the great honor due to Dr. Morton, greater honor is due to another dentist. For it is true, and is now being admitted, that Dr. Morton but traveled in another path, though further than had been traveled two years before to his own knowledge, by the true and original discoverer of anesthesia, from whom he derived his incentive, the late able, but less persevering and obstecle-overcoming dentist of Boston, Dr. Horace Wells.

If we grant that the whole includes all parts, though one part may be so brilliant as to overshadow the rest; if we grant that an inventor of something entirely new is entitled to credit superior to him who invents an improvement or modification, even though the latter may be better; if we grant that the discoverer of a great truth or principle in nature is greater than the one who, following in the same lines, by using other agents or methods more fully or successfully demonstrates the truth or principle—we must admit that the greater honor is due to Dr. Wells—provided it is true that, in 1844, two years previous to Dr. Morton's discovery, Dr. Wells did intelligently and publicly, with full appreciation of the phenomena, perform painless operations in dentistry and surgery by the administration of nitrous oxid gas, given for that specific purpose. I think that history bearing this out is too explicit, too minute, and too reliable, to render this statement debatable. Let us inquire what are admitted facts. It is accepted by every one as proven:

1st. That Mr. G. Q. Colton did give an entertainment in Hartford, in 1844, by the exhibition of laughing gas, for amusement.

2d. That Dr. Horace Wells was present in the audience.

3d. That Mr. Samuel A. Cooley, in his antics while under the influence of gas, injured his leg quite severely.

4th. That on his recovery from the effects of the gas, Mr. Cooley was surprised at the injury, and said he felt no pain.

5th. That Dr. Wells concluded from this that the gas would be useful in extracting teeth, and so expressed himself.

6th. That Dr. Wells did put his inference to proof by an actual experiment upon himself—Mr. Colton giving the gas and Dr. J. M. Riggs extracting a tooth.

7th. That his object, stated beforehand, was to ascertain by trial whether such exhibition of the gas would render tooth-extraction painless.

8th. That Dr. Wells did state at once that the operation was painless.

9th. That Dr. Wells did many times give the gas for painless operations in Hartford, both in dentistry and surgery.

10th. That Dr. Wells had such faith in his discovery that he came to Boston in the winter of 1844-45, and was introduced to the hospital surgeons by his former pupil, Dr. Morton, and did administer the gas at the Massachusetts General Hospital, for an operation, with the express pre-stated purpose of demonstrating that operations could be painlessly performed.

11th. That the exhibition at the hospital was only a partial success.

12th. That the students derided him, and that he went home to Hartford disappointed and disheartened.

13th. That Dr. Morton had some knowledge of these facts--anterior to 1846.

14th. That the use of the gas as an anesthetic was discontinued in Hartford and elsewhere after Dr. Wells' death, and the successful demonstration of the efficacy of ether for the same purpose.

15th. That in 1862 the use of gas for anesthetic purposes was resumed, and that it has since been proven throughout the whole world to be a safe and reliable agent for that purpose by many millions of exhibitions.

The discovery of the efficacy of chloroform in 1847, and its rapid spread over Europe to the almost total exclusion of ether, gave such fame to its discoverer, Dr. Simpson, afterward Sir James Y. Simpson, that for many years in Europe he was generally reputed to be the discoverer of anesthesia.

These two anesthetic agents had the field almost exclusively for about fifteen years until the revival of nitrous oxid in 1862, so that most naturally the agent used and the resulting anesthesia became synonymous terms in the general understanding. It is not strange that the neglected and forgotten nitrous oxid during this long period should have had as companion in its oblivion the name and fame of Horace Wells. But its revival in 1862, and its general and successful adoption throughout the world, demonstrates that it is second to no other agent, and proves that its short use, before ether eclipsed it, was due to fortuitous circumstances in no way detracting from the merit rightfully belonging to the diffident, sensitive, generous and noble man, who so soon after, disappointed and with unsettled intellect, met his tragic death, but whose memory is still green in the field of his labors and in the hearts of his fellow-citizens.

With commendable regard for truth and history, his city and State have testified to his worth and achievement by erecting his statue in enduring bronze.

A very beautiful monument, commonly designated as the Ether Monument, was erected in the Public Garden,

Boston, in 1867, by the munificence of a private citizen. The inscriptions read:

"In gratitude
For the relief
Of human sufferiug
By the inhaling of Ether
A Citizen of Boston
Has erected this monument
A. D. 1867."

---

"To commemorate
The discovery
That the inhaling of Ether
Causes insensibility to pain
First proved to the world
at the
Mass. General Hospital
in Boston
October, A. D. 1846."

At the time of the erection of this memorial, many of those active in the first exhibition of ether were still living and were friends of the donor. It is presumable that great care was exercised to make the inscriptions impregnable to criticism. Two things should be especially noted—the absence of any name and the restriction of credit to ether alone.

However we may view the question as to the right to first place of honor for Wells or Morton, we can congratulate the profession that both were dentists, and that this greatest boon of the ages came from our ranks. So great an authority as Lecky says, in his "History of European Morals," "It is probable that the American inventor of the first anesthetic has done more for the real happiness of mankind than all the philosophers from Socrates to Mill."

While in the following decade, 1850 to 1860, colleges, magazines and associations multiplied and jointly contributed to bring the profession more and more in touch with progressive thoughts and truths, the most distinctive discovery of the decade and most momentous in its influence was that property of gold, which, previously considered detrimental, was now to be welcomed as its most valuable characteristic—cohesion. The introduction of crystal gold and the discovery of the cohesiveness of freshly annealed foil laid the foundation for the new era in operative dentistry. Let us never forget that while others claimed the latter discovery and doubtless had known of it and availed themselves of it for some time, Dr. Robert Arthur lost no time in freely sharing his discovery, as soon as made, with the whole profession. He thus achieved a distinction of which others have never been able to deprive him.

The descriptions and illustrations of operations with crystal gold in the essay of that venerable and respected Nestor still with us, Dr. W. H. Dwinelle, published in 1855, might still answer for an essay of to-day. Here was the renaissance of operative dentistry. Here was the dawn of the new era of restoration; the parting line between antique mutilation and disfiguration and the subsequent devotion to beauty and typal form. It was the first great advance in practice. It was but natural that it should soon be supplemented by improved instruments, the mallet, the rubber-dam and the engine. How great a revolution has resulted from these instruments and appliances none can fully realize, except those of us who have been long enough in practice to remember the struggles necessary in the old era. However radical or conservative are the views we hold to-day, there can be no question of the tremendous shaking up of the profession in its thoughts and practice which resulted from these innovations.

While most of the appliances just mentioned came in during the decade 1860 to 1870, they do not constitute, it seems to me, the distinctive advance of that decade. There had been a disease of the mouth, which, up to this time, had been either unrecognized or regarded universally as incurable. It had from a remote period been described in the books as scurvy of the gums, or some such term, and had been treated only by washes or medication. It was considered inevitable and irremediable that sound teeth should be lost, self-extracted. A prophet arose who taught that such deplorable conditions were always preventable if taken in time, and frequently remediable by surgical treatment when the disease had made quite extensive inroads. He was received as prophets usually are, except by a few who early became his disciples. He was not a profound physiologist or pathologist, and did not present a theory or description which met the approval of experts; but he had lived many years, was a man of observation and reflection, and while not scholastic and correct on every point, his observations had been clear and his deductions in the main correct, so that the treatment which he was the first to bring out and demonstrate is even at this day, after so many of our best pathologists have devoted much time to the study of the disease, accepted as the foundation of all treatment.

The men who knew him, saw him operate, were taught by him, and were successful in following his methods, were wont to call the disease after his name. This was not correct, we know, for he originated only a treatment and did not describe a disease; but the fact remains, and there are enough living to testify to it, that as a result of his life and efforts, of the seed which he planted, a dire disease has been robbed of its terrors, the profession has been stimulated throughout the world to study its etiology and progress, and the premature loss of teeth from this disease is no longer considered providential or respectable.

While operative dentistry has continued to ride constantly upon a flood-tide of progress and improvement, prosthetic dentistry has had its ebbs and floods. Sixty years ago the great mass of the profession were unskilled as operators, but fairly skilled as plate workers. They could not save teeth, but they could replace their loss. In plate work the culmination of prosthetic skill and artistic production came with the invention and perfection of porcelain or continuous gum. After the introduction of vulcanite, the general disuse of metals made laboratory skill of little value, and hence it was neglected or ignored in the preparatory training of the student. The manufacturers supplied a great variety of instruments, so that the forging, shaping, and tempering of instruments became almost a lost art. The ease and facility of working of vulcanite not only called for little ingenuity and skill, but so obliterated the distinctions that the novice, after a few weeks of instruction and practice, could compete with the most experienced, and this important and most beneficial branch became the refuge and ally of incompetence and quackery. The evils resulting from the wholesale extraction of good teeth were most deplorable and cannot be estimated.

There has been grave doubt whether vulcanite in dentistry has been a blessing or a curse to the world. A superficial observer might contend that when organs so important to health were lost, it was a blessing that substitutes could be within the reach of all but those in abject poverty. But we know to what a shameful extent the Harpies in our number, by appeals to cowardice and cupidity, despoiled the mouths of the confiding public of millions upon millions of strong and healthful teeth to make place for their bungling, disfiguring, and filthy substitutes. It did seem for a time that the whole profession would be engulfed in a sea of obloquy. But the reaction, happily came, the tide turned, and we can congratulate ourselves and the world that the danger is fast disappearing.

The increase in the number and the constant elevation of the standards of the colleges year by year, raised the ratio of the educated; the periodical literature was more generally taken and read; societies multiplied and did most valliant missionary work; Codes of Ethics were adopted and enforced; laws were passed for the protection of society, which, while licensing all in practice to continue practicing, irrespective of their knowledge or skill, raised a barrier against the admission to practice of the ignorant and incompetent. From all these and other causes the tone ot the profession was gradually raised, and juster and broader views of what was right and best for the patient became more and more prevalent.

But the cause more important than any or perhaps all of the foregoing for the increase of laboratory skill and the retention of teeth and roots is to be found in the invention of the modern artificial crown and its corrollary, the bridge.

This is the distinctive improvement of the past twenty years. Within that period more than one hundred different crowns and bridges have been invented and published.

The result has been two-fold. It has made laboratory skill of more importance and value to the dentist than ever before, and it has arrested the great "slaughter of the innocents" by making the retention of the roots of teeth in the mouth, obligatory.

At various periods the separation of the two branches of practice has been urged by prominent men of each branch, but by these inventions the two branches have been bound together in bonds which seem indissoluble.

The chief drawback to perfection in the past has been the inability of our art, however skillful, to permanently save some teeth. The inherent defects of structure or of surroundings made the best operations but temporary, and these teeth had ultimately to be lost and substitutes applied. Now, after all the worst has happened, the root is still of inestimable value for crowning. The invention seems to place a climax upon our art.

In recent years there has been an increasing interest in the deeper causes of physiological function and of pathological departure from normality. Histological investigations have been pursued with great enthusiasm and thoroughness, and the advances in other departments of microscopic research are largely due to methods which were first devised and employed in the study of dental tissues by ingenious dental microscopists. These fields of research are inviting to the student, but demand great courage and self denial when cultivated by those whose labors are severe and fatiguing in the daily routine of the office. We rejoice that there are so many dentists throughout the world who are devoting their energies, after the day's work is done, to these problems. We shall be encouraged as well as edified through the week by the display of results already achieved.

How crude and speculative seem the theories of dental caries which obtained less than a score of years ago when contrasted with the brilliant demonstrations of the renowned American professor of Berlin, founded upon patient and protracted investigation after the most approved modern scientific methods. Though from unavoidable circumstances detained at home, he has shown his interest and co-operation by forwarding a paper. There are many others whose fame is not bounded by their vocation or their country. They are known to the world as scientists and cosmopolitans. However skillful and judicious a dentist may be as an operator, this sphere of his usefulness is limited in space and not far-reaching, while those men are working for mankind at large and for succeeding generations. In view of past victories may we not confidently expect that the etiology of other still obscure diseases, like erosion and pyorrhea, may be solved so that we can either prevent or successfully treat them?

Let us not grudge explorers of the unknown their only recompense, the meed of praise and applause for what they have done and will do for the profession and for humanity.

I am conscious that in this review of the past I have failed to do justice to the subject.

Time is so limited, and the field so rich and extensive, that I have felt constrained to select for elaboration only those facts and movements which seemed to have the greatest influence.

Joy and comfort have been brought to many repulsive or speechless sufferers by the improved appliances for the restoration of feature or of function resulting from malformation or disease. Orthodontia might be called a new art, from the great advance in knowledge of it and of the methods and appliances for correction, and recovery of usefulness and beauty.

But the most prominent of the prodigal topics which I have ignored is probably plastics, the increasing use of which has wrought a great change in practice, and been of incalculable benefit to humanity. From the time of the memorable controversy which caused the disruption of the first dental society to the present period, this subject has engrossed much time and talent in our societies and literature.

The so-called "New Departure" arrested the attention of every reader from its novelty and from its claim to be founded upon strict scientific experimentation. Laboratory investigations are valuable, if conducted upon strictly scientific methods; but is not a theory based upon the observation of the phenomena as they are presented in the oral cavity also essentially scientific, if the observer is competent and the series of observations have been correctly performed? In either case accuracy of observation in connection with a clear appreciation of exactly what constitutes the scientific method, and the intelligent application of that method in the elucidation of the point at issue, is what is needed. I do not, however, attempt to discuss this subject, but only refer to it as one which has had a great influence in stimulating thought, making us closer observers, and modifying practice.

The theory of the bacterial origin of disease, and antisepsis, have engrossed much study and enthusiasm. It seems that we were on the eve of a solution of all etiology by the isolation of the specific pathogenic germ of every disease, and that to prevent or cure all that was necessary was to ascertain and administer the proper germicide. Clinical records, however, have failed to sustain the ardent expectation of the more sanguine. The germicide has taken a place subordinate to a strict observance of absolute cleanliness, and we see acknowledged the importance of *vis vitæ* as a factor in securing immunity from the attacks of bacteria everywhere present.

Light, however, is being shed upon many mysterious bacterial phenomena by a more thorough acquaintance with ptomaines, leccomaines, and the extractives which are toxic and anto-intoxicants. Fortunate practical results have come in the character of a great reform in the care of instruments and the sterilization of everything connected with operative work.

All through the years there has been an underlying hope that improvement of tissue and greater resistive power would result from advances in prophylaxis. Apart from the improved conditions resulting from changed manipulation like contouring, or greater care in cleanliness, little has been accomplished in purely dental prophylaxis. Special feeding for the teeth or treatment in the line of therapeutics has not rewarded patient trial.

The tendency of prophylaxis to-day is to develop along physiological lines by more special and minute observance of the laws of hygiene. Proper food, its preparation for assimilation, out-door exercise, in a word, rational methods of living, are no more important for the preservation of health than for the building up of tissues throughout the whole body, of such perfectness of structure and function as to be able successfully to resist deleterious attacks. Thus viewed, prophylaxis has made great strides, in which the teeth have shared as part of the general economy. These topics and many others merit more extended consideration as factors of progress.

While I have refrained from specific mention, with two exceptions, of the living exponents of progress, I cannot close without a brief reference to the many upon our Roll of Honor. It would be pleasant to name them, review their lives, and pronounce their eulogy. "They builded better than they knew," and the profession and the world are their debtors. Our duties are less onerous, our paths are more pleasant, our position among men more honorable, and the health, comfort, and relief from suffering of mankind greatly enhanced as a result of the lives and the labors of the noble men who, in the language quoted before, were "impressed with the belief that the resources of the science were by no means developed, and that it was their duty to devote a portion of their time and energy to the work."

Some of these apostles of progress are known to us only by their records, but the form, the features, the voice, the presence of some of them are fresh in the memory of many here present.

Can we doubt their approval to-day of our assemblage, or of their presence among us and their benediction? This Congress is but a continuation upon the same lines, or a culmination of their patient, devoted, self-sacrificing work for God and man.

Under the authority of the trust committed to us by the dental profession of America, we are now assembled and organized as a World's Dental Congress, to advance the interests of dentistry throughout the world. Science and art, twin outgrowths of mentality, should know no boundaries, nor should there be any schools of thought, treasure-houses of knowledge, or gymnasia for training to usefulness, with barriers founded upon race, creed or nationality, where the health of humanity is involved. As dentists we meet here to-day, brothers of one family, with common interests, mutual respect, and unity of aspirations and expectations. With infinite care and labor the banquet has been prepared, and we are invited to partake of the ripest fruits of professional culture which could be gathered from every quarter. Let us partake joyfully, not for the pleasure of the day alone, but from the consciousness that each feast brings with it the earnest of greater strength to-morrow.

In my official capacity I bid you all welcome—a welcome to every American dentist, who has come with comparative ease, but an especial welcome to those who have traveled long and far, from foreign climes and over broad seas, bringing with them the choice perfumes and rare products of a different soil from ours, but which are indispensible to the adornment and complete furnishing of the universal banquet of good things for us and for mankind. That these may find their visit both pleasant and profitable is the wish of every one of their American hosts. And finally, let us all improve the opportunities presented, that this week may be so full of inspirations and valuable results that it shall be a mile-stone in the march of dental professional progress.

---

ARTICLE II.

